# 16S rRNA gene amplicon dataset of prokaryotic communities from a subantarctic marine ecosystem: Ushuaia Bay and surrounding waters

**DOI:** 10.1016/j.dib.2020.106171

**Published:** 2020-08-12

**Authors:** Clara Natalia Rodríguez-Flórez, Andrea Malits, Mariana Lozada

**Affiliations:** a Laboratorio de Oceanografía Biológica (CADIC-CONICET), Ushuaia, Argentina; b Laboratorio de Microbiología Ambiental (IBIOMAR-CONICET), Puerto Madryn, Argentina

**Keywords:** Bacteria, Archaea, Seawater, Subantarctic, 16S rRNA, Gene amplicon sequencing

## Abstract

We provide a 16S rRNA gene dataset of prokaryotic assemblages of a subantarctic marine ecosystem. Samples were collected at 2 stations (one near Ushuaia Bay and the other close to Bridges islands in the Beagle Channel). At each station, 2 depths (subsurface and bottom waters) were sampled in february, march, may and september during 2018, giving a total of 10 samples. 16S rRNA gene amplicon sequencing (V4 hypervariable region) was performed with the Illumina MiSeq platform. A total of 1116 amplicon sequence variants (ASVs) were recovered from the dataset. The sequences were taxonomically assigned to Alphaproteobacteria (23 ± 2%, mean ± standard error), Gammaproteobacteria (17 ± 1.5%), Flavobacteriia (8 ± 2%), Deltaproteobacteria (3.7 ± 0.5%), Acidimicrobiia (1.7 ± 0.1%), Planctomycetia (1.9 ± 0.4%), and AB16 group (1.7 ± 0.3%). Sequences affiliated with Archaea were abundant, reaching one third of analyzed sequences, mainly Thaumarchaeota (22 ± 3%), and Thermoplasmata (10 ± 1%). Together, sequences assigned to all these groups accounted for more than 90% of the sequences. This dataset constitutes a valuable resource for future scientific research aiming to unveil the role of these communities in ecosystem services such as carbon and nutrient cycling, and pollutants degradation. This will turn into benefits for future environmental monitoring and preservation actions, considering the tangible heritage of Ushuaia Bay and surrounding waters.

**Specifications Table**

**Table:** 

**Subject**	Environmental Science (General)
**Specific subject area**	Marine microbial diversity and ecology
**Type of data**	fastq file
**How data were acquired**	16S rRNA gene amplicon sequencing Instruments: Illumina MiSeq platform, QIIME2 software
**Data format**	Raw Filtered Analyzed
**Parameters for data collection**	Conditions considered for data collection were: seawater samples from subsurface and bottom, at two stations: one in Ushuaia Bay and the other in surrounding waters. *Subsurface waters:* -20m. *Bottom waters:* ∼ -120m (Ushuaia Bay station) and ∼-148m (surrounding waters station).
**Description of data collection**	Seawater samples were collected from a boat in Niskin bottles, prefiltered and passed through a Sterivex filter (0.2µm). Metagenomic DNA extraction was performed from filters, and after PCR amplification of 16S rRNA gene V4 hypervariable region, amplicon sequencing was done on Illumina MiSeq platform. Preprocessing and taxonomic profiling was performed in QIIME2.
**Data source location**	City/Region: Ushuaia/Tierra del Fuego Country: Argentina Latitude and longitude: *Ushuaia bay station:* 54° 48.728´S, 68° 14.388 W, close to Ushuaia city (Tierra del Fuego, Argentina). *Surrounding waters station:* 54° 52.939´S, 68° 10.927´W, close to Bridges islands and near kelp forest, in the Beagle Channel. Maritime traffic: the sampling stations are located along a sea route. Possible environmental impact. High nutrient and organic matter load to Ushuaia Bay through glacial runoff and urban and industrial activities.
**Data accessibility**	The sequence data from this work is available at the NCBI Genbank Sequence Read Archive (SRA) *as Bioproject ID:* PRJNA622742 *and SRA accession no. PRJNA637656.* Repository name: Genbank Sequence Read Archive Data identification number: PRJNA637656 (Bioproject) SRR11941686-SRR11941695 (Runs) Direct URL to data: Bioproject: https://www.ncbi.nlm.nih.gov/Traces/study/?acc=PRJNA637656&o=acc_s%3Aa Runs: https://trace.ncbi.nlm.nih.gov/Traces/sra/?run=SRR11941686 https://trace.ncbi.nlm.nih.gov/Traces/sra/?run=SRR11941687 https://trace.ncbi.nlm.nih.gov/Traces/sra/?run=SRR11941688 https://trace.ncbi.nlm.nih.gov/Traces/sra/?run=SRR11941689 https://trace.ncbi.nlm.nih.gov/Traces/sra/?run=SRR11941690 https://trace.ncbi.nlm.nih.gov/Traces/sra/?run=SRR11941691 https://trace.ncbi.nlm.nih.gov/Traces/sra/?run=SRR11941692 https://trace.ncbi.nlm.nih.gov/Traces/sra/?run=SRR11941693 https://trace.ncbi.nlm.nih.gov/Traces/sra/?run=SRR11941694 https://trace.ncbi.nlm.nih.gov/Traces/sra/?run=SRR11941695

**Value of the Data**

• This is the first checklist of amplicon sequence variants and their taxonomic classification from Ushuaia Bay and surrounding waters in the Beagle Channel, a subantarctic marine environment exposed to anthropogenic impact and suffering rapid shifts due to climate change.
• This first 16S rRNA gene profiling of prokaryotic assemblages of a subantarctic marine ecosystem is a valuable resource for the scientific community as well as for institutions performing environmental monitoring and preservation actions.
• This dataset is useful for upcoming scientific research covering the role of these communities in ecosystem services.

## 1 Data description

**Fig. 1:**
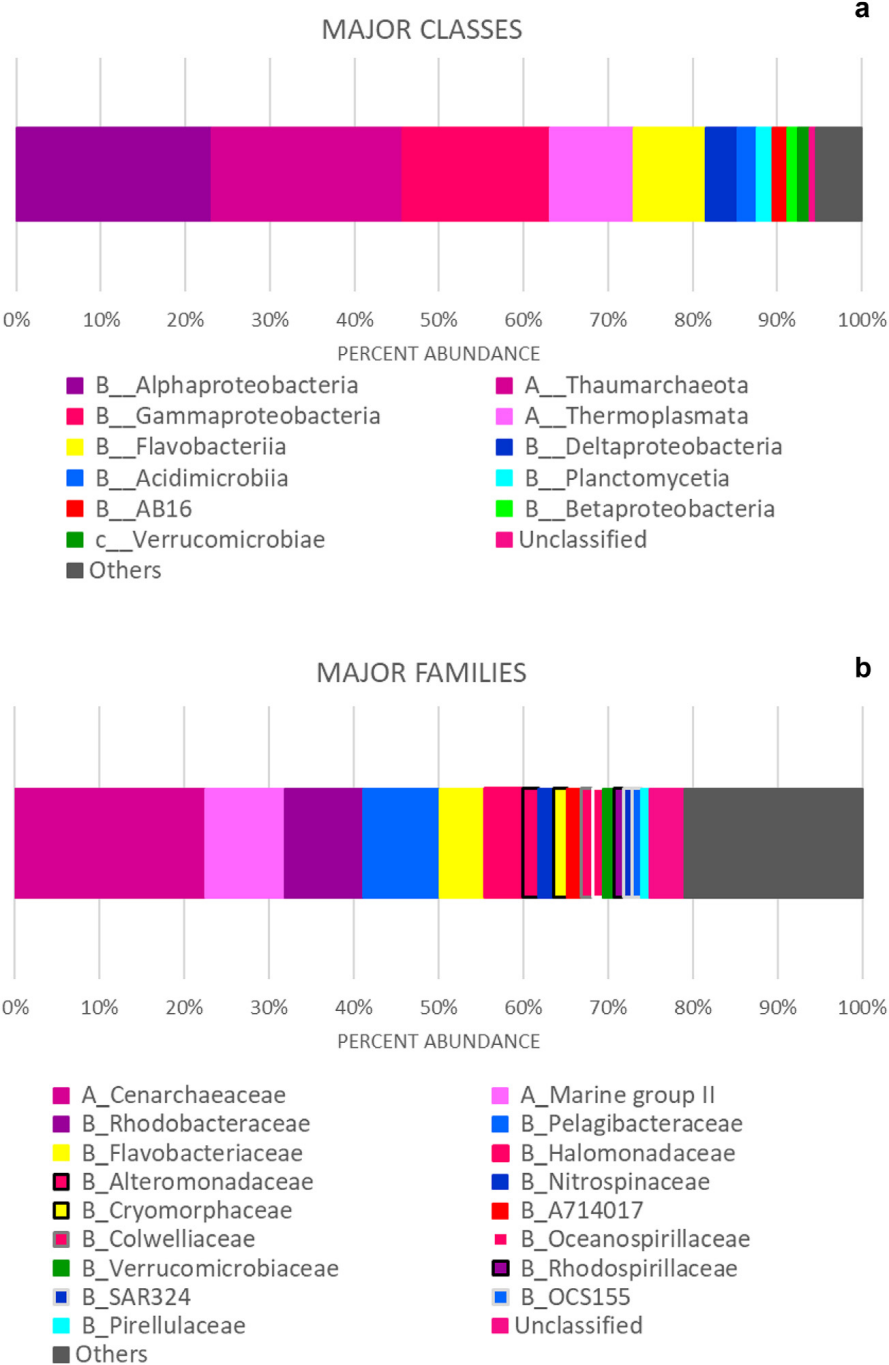
Percent abundance of major (a) classes and (b) families, identified in prokaryotic communities from Ushuaia Bay and surrounding waters in the Beagle Channel. Only classes and families with more than 1% relative abundance are shown. A_: Archaea. B_: Bacteria.

The raw sequencing dataset contained 550,960 sequences from a total of 10 samples. After preprocessing, the dataset contained 302,986 sequences, 150 bp on average. **A total of 1116 amplicon sequence variants (ASVs) were recovered from this environment.** Of the total analyzed sequences, **68 ± 3 % corresponded to Bacteria** (mean ± standard error) and **32 ± 3 % to Archaea. The major phyla** (out of 24, B__: Bacteria and A__: Archaea) **were**: B__*Proteobacteria* (45 ± 2 %), A__*Crenarchaeota* (22 ± 3%), A__*Euryarchaeota* 10 ± 1%, B__*Bacteroidetes* 9 ± 2% and B__*Planctomycetes* 4 ± 0.6%. **The most abundant classes (out of 40) were**: B__*Alphaproteobacteria* 23 ± 2%, A__*Thaumarchaeota* 2 ± 3%, B__*Gammaproteobacteria* 17 ± 1%, A__*Thermoplasmata* 10 ± 1%, B__*Flavobacteriia* 8 ± 2%, B__Deltaproteobacteria 4 ± 0.5%, B__Acidimicrobiia 2 ± 0.1%, B__Planctomycetia 2 ± 0.4%, B__AB16 1.71 ± 0.2%. **The most representative orders (out of 60)** were: A__Cenarchaeales 22 ± 3%, B__Oceanospirillales 10 ± 0.7%, A__E2 10 ± 1%, B__Rhodobacterales 9 ± 2%, B__Rickettsiales 9 ± 1%, and B__Flavobacteriales 8 ± 2%. **The major families (out of 78) were:** A__*Cenarchaeaceae* 22 ± 3%, A__*Marine group II* 9 ± 1%, B__*Rhodobacteraceae* 9 ± 2%, B__*Pelagibacteraceae* 9 ± 1%, B__*Flavobacteriaceae* 5 ± 1%, B__*Halomonadaceae* 4 ± 0.6%, B__*Alteromonadaceae* 1.8 ± 0.5% and B__*Nitrospinaceae* 1.8 ± 0.3% (Fig. 1).

**The major genera (out of 82) were**: A__*Nitrosopumilus* 22 ± 3%, B__*Pelagibacter* 7 ± 1%, B__*Candidatus Portiera* 4 ± 0.6%, B__*Sulfitobacter* 4 ± 1%, 1.7 ± 0.3%, B__*Colwellia* 1.4 ± 0.7%, B__*Polaribacter* 1.3 ± 0.4% and B__*Glaciecola* 0.9 ± 0.5%.

**Supplementary File 1.** Bioinformatic script used to preprocess the raw sequences. QIIME2 v 2019.1 (https://qiime2.org/) was used to process the data.

## 2 Experimental design, materials and methods

Seawater was collected in Niskin bottles (5L) and prefiltered by a mesh of 213 µm after which each water sample (∼4L) was concentrated in a Sterivex filter unit of 0.22 µm (Millipore). The Sterivex units were stored without excess liquid into sterile sampling bags (Microclar) at -20°C, until cell lysis and nucleic acid extraction. The sampling took place during February (2 samples), March (1), May (4) and September (3) 2018.

Cell lysis and nucleic acid extraction were carried out following the protocol modified from Somerville and collaborators [1]. DNA quantification was done using Lambda Phage DNA (Promega). Samples were sequenced at INDEAR (Argentina). Quantification of initial DNA samples was performed using the Quant-iTTM PicoGreen® DNA Assay Kit (Invitrogen). Amplicons of V4 region were obtained by using primers 515F (GTGCCAGCMGCCGCGGTA)[2] and 806R (GGACTACNVGGGTWTCTAAT)[3]. A second round of PCR was performed with standard Illumina barcodes and adapters. Libraries were pooled at the same concentration and 1ul of the pool was run in the 2100 Bioanalyzer (Agilent Technologies) using the DNA 12000 chip Kit. The library pool was quantified using DeNovix. Libraries were sequenced on an Illumina MiSeq platform.

The generated reads were preprocessed with the QIIME2 package [4]. Briefly, demultiplexed paired-end reads were trimmed to 150 bp, merged and the resulting sequences were denoised in Deblur [5] in QIIME2 environment, in order to identify amplicon sequence variants (ASV). ASVs were taxonomically classified in QIIME2 using Greengenes classifier (https://data.qiime2.org/2019.1/common/gg-13-8-99-515-806-nb-classifier.qza). The resulting feature table and taxonomic assignments were analyzed in QIIME2 and phyloseq [6].

**Supplementary File 1:** Bioinformatic script used in QIIME2.

## Ethics statement

The work did not involve the use of human subjects or animal experiments.

## Declaration of Competing Interest

The authors declare that they have no competing financial interests or personal relationships which have, or could be perceived to have, influenced the work reported in this article.

## Appendix Supplementary materials

Image, application 1

Image, application 2
